# Autophagy regulates vinorelbine sensitivity due to continued Keap1-mediated ROS generation in lung adenocarcinoma cells

**DOI:** 10.1038/s41420-018-0098-6

**Published:** 2018-09-12

**Authors:** Yan-Wei Wu, Chiou-Feng Lin, Yee-Shin Lin, Wu-Chou Su, Wei-Hsin Chiu

**Affiliations:** 1 Department of Medicine, College of Medicine, National Cheng Kung University, Tainan, Taiwan; 2 Graduate Institute of Medical Sciences, College of Medicine, Taipei Medical University, Taipei, Taiwan; 3 Center of Infectious Disease and Signaling Research, National Cheng Kung University Medical College, Tainan, Taiwan; 4 Department of Internal Medicine, National Cheng Kung University Hospital, College of Medicine, National Cheng Kung University, Tainan, Taiwan

## Abstract

Autophagy is one of the induced mechanisms in metastatic cancer to escape death due to starvation, hypoxia, metabolic stresses, chemotherapy, and radiation. Some publications have revealed that chemotherapy combined with autophagy inhibitor will overcome drug resistance. We modified AS2 cells with PTEN overexpression, mTOR knockdown, or Keap1 knockdown, and made modification of A549 cells with PTEN knockdown, Atg5 knockdown, and Keap1 overexpression. Our study was aimed toward an exploration of how autophagy modulates Keap1, ROS generation, and vinorelbine-induced apoptosis in these cell lines. We found that lung cancer PC14PE6/AS2 (AS2) had higher mTOR and Akt and also lower PTEN expression than A549 cells. Descended autophagy was demonstrated with more decreased p62 accumulation and LC3 II conversion in AS2 cells as compared to A549 cells. The A549 cells had lower Keap1/Nrf2 and more active anti-oxidant response element (ARE) activity than the AS2 cells. We modified AS2 cells with PTEN overexpression, mTOR knockdown, Keap1 knockdown, and revealed amplified p62 and LC3 expression accompanied with decreased Akt, Keap1, ROS, and vinorelbine-induced apoptosis. Declined p62, LC3 expression were accompanied with increased Akt, Keap1, ROS, and vinorelbine-induced apoptosis after modification of A549 cells with PTEN knockdown, Atg5 knockdown, and Keap1 overexpression. Keap1 overexpression lowered ARE levels in A549 cells, and ARE level exhibited up-growth in Keap1 knockdown AS2 cells. The autophagy inhibitor caused more ROS generation and vinorelbine-induced apoptosis in the A549 and CL1-5 cells. According to these findings, autophagy regulates vinorelbine sensitivity by continuing Keap1-mediated ROS generation in lung adenocarcinoma cells.

## Summary

It needs more evidence to prove that chemotherapy combined with autophagy inhibitor will overcome drug resistance. The pilot study demonstrated a VNR-sensitive strategy in lung adenocarcinoma cells, by which regulators of autophagy modulated Keap1-mediated ROS generation and VNR-induced apoptosis.

## Introduction

Lung cancer causes many deaths worldwide, and there are increasing numbers of publications exploring the relationship between lung cancer and autophagy. Autophagy has a dual role in lung cancer, including being an inhibitor of initial oncogenesis and then facilitating tumor progression^1^. Autophagy is well known as a catabolic process, so recycling cellular components and damaged organelles all require autophagy to maintain homeostasis^2^. In eukaryotic cells, autophagy can be divided into three major intracellular pathways, including macroautophagy, microautophagy, and chaperone-mediated autophagy (CMA)^3^. Reactive oxygen species (ROS) and reactive nitrogen species (RNS) regulate intracellular signal transduction and sustain autophagy^4^. p62 is an autophagy adaptor protein to regulate the degradation of the proteins accumulated in inclusion body^5^.

The tumor suppressor gene *phosphatase and tensin homolog (PTEN)* was first identified in 1997, and a previous study revealed that 74% of protein expression is decreased or lost in lung cancer^6^. Because PTEN negatively regulates the PI3K-Akt-mTOR pathway, some targeted therapy has been developed to enhance its expression in tumors with PTEN loss^7^. Besides, PTEN increases autophagy in glioma cells, and the PI3K-Akt-mTOR pathway can be used to regulate autophagy^8^. Genetic or pharmacologic inhibition of mTOR, a serine/threonine kinase, induces autophagy in yeast^9,10^. Vinorelbine (VNR) was developed in 1979 and was shown to be a semi-synthetic second generation vinca-alkaloid^11^. In our previous study, VNR cause aberrant ROS-mediated JNK activation, Mcl-1 downregulation, DNA damage, mitochondrial dysfunction, and apoptosis in lung adenocarcinoma AS2 cells^12^. Compared with AS2 and CL1-0 cells, apoptotic analysis showed that both A549 and CL1-5 cells were VNR-resistant, while these cells highly expressed glucosylceramide synthase (GCS) at the protein level^13^.

The Keap1-Nrf2 pathway regulates cytoprotective responses to ROS-induced stress^14^. In a canonical pathway, modification of Kelch-like ECH-associated protein 1 (Keap1) inhibits nuclear factor erytheroid-derived-2-like 2 (Nrf2) ubiquitylation and stabilizes Nrf2. Keap1induces Nrf2 accumulation in cytosol and then translocates into the nucleus. Nrf2 binds to genes containing antioxidant response elements (AREs) and activates transcription^15^. Nrf2 facilitates cancer chemoresistance and enhances tumor growth^16^, and Keap1 degradation is mediated by autophagy for the maintenance of redox homeostasis^17^.

PTEN and mTOR expression were previously detected in our experiment. We thus aim to explore whether autophagy regulates vinorelbine sensitivity through Keap1/Nrf2-mediated ROS generation in lung cancer cells.

## Materials and methods

### Cell culture and reagents

The human lung adenocarcinoma PC14PE6/AS2 (AS2) cell line was established from ascites generated from PC14PE6 cells (a gift from Isaiah J. Fidler; MD Anderson Cancer Center, Houston, TX, USA) in nude mice, and the protocol was described previously^18^. The CL1-0 and CL1-5 cells were generously provided by Dr. Pan-Chyr Yang (Department of Internal Medicine, National Taiwan University Hospital). AS2 and human lung adenocarcinoma A549 (CCL185, ATCC), CL1-0, and CL1-5 cells were grown on plastic in Dulbecco’s modified Eagle’s medium (Gibco-BRL; Grand Island, NY, USA) with L-glutamine and 15 mM HEPES, supplemented with 10% fetal bovine serum (Gibco-BRL), 100 units of penicillin, and 100 µg/ml streptomycin and maintained at 37 °C in 5% CO_2_. All cell lines were sub-cultured no more than 3 months in our lab. Other chemical drugs used for cell culture and autophagy inhibitor 3-methyladenine (3-MA) were purchased from Sigma-Aldrich (St. Louis, MO, USA). The vinca alkaloids VNR was purchased from Sigma-Aldrich. For the luciferase reporter assay, the cells were transiently cotransfected using QIAGEN with Nrf2 and a Nrf2 promoter-driven luciferase reporter (0.2 mg), the Nrf2 and Nrf1 bound to antioxidant response elements (AREs), and 0.01 mg Renilla luciferase-expressing plasmid (pRLTK; Promega). 24 h after transfection, the cells were treated with IFN-γ for 1 h, lysed, and then harvested for the luciferase and Renilla measurements using a luciferase assay system (Dual-Glo; Promega). For each lysate, the firefly luciferase activity was normalized to the Renilla luciferase activity to assess transfection efficiencies. The β-actin antibodies and horseradish peroxidase-conjugated and Alexa 488-conjugated anti-rabbit IgG were obtained from Chemicon International (Temecula, CA, USA).

### Cell apoptosis assays

To observe nuclear condensation, 4’,6-diamidino-2-phenylindole (DAPI; Sigma-Aldrich)-stained cells were observed using a fluorescence microscope (IX71; Olympus, Tokyo, Japan). Cell apoptosis levels were analyzed using nuclear propidium iodide (PI; Sigma-Aldrich) staining and flow cytometry (FACSCalibur; Becton Dickinson, San Jose, CA) with the excitation set at 488 nm and emission detected with the FL-2 channel (565–610 nm). The distribution of the cells in the different phases of the cell cycle was calculated using MetaMorph software (Molecular Devices, Downingtown, PA, USA). For the apoptosis analysis, the samples were analyzed using CellQuest Pro 4.0.2 software (Becton Dickinson), and quantification was performed using WinMDI 2.8 software (The Scripps Institute, La Jolla, CA, USA). Apoptosis levels are reported as the percentage (%) of cells in the sub-G_1_ phase.

### Western blot analysis

Harvested cells were lysed with a buffer containing 1% Triton X-100, 50 mM of Tris (pH 7.5), 10 mM of EDTA, 0.02% NaN_3_, and a protease inhibitor cocktail (Roche Boehringer Mannheim Diagnostics, Mannheim, Germany). Following one cycle of freeze-thaw, the cell lysates were centrifuged at 10,000×*g* at 4 °C for 20 min. and then boiled in sample buffer for 5 min. The proteins were then subjected to SDS-PAGE and transferred to a PVDF membrane (Millipore, Billerica, MA, USA) using a semi-dry electroblotting system. After blocking with 5% skim milk in PBS, the membranes were incubated with a 1/1000 dilution of primary antibodies at 4 °C overnight. The membranes were then washed with 0.05% PBS-Tween 20 and incubated with a 1/5000 dilution of horseradish peroxidase-conjugated secondary antibodies at room temperature for 1 h. After washing, the membranes were soaked in ECL solution (PerkinElmer Life Sciences Inc., Boston, MA) for 1 min, and then exposed to film (BioMax; Eastman Kodak, Rochester, NY, USA). The relative optical density (OD) of the signal protein was quantified using ImageJ software (version 1.41o) from W. Rasband (National Institutes of Health, Bethesda, MD, USA).

### Transfection

Keap1 expression was silenced using commercialized siRNA (sc-43878) from Santa Cruz Biotechnology (Santa Cruz, CA, USA). Transfection was performed using electroporation, with a pipette-type microporator (Microporator system; Digital Bio Technology, Suwon, Republic of Korea). After transfection, the cells were incubated for 18 h in RPMI medium at 37 °C before infection. A nonspecific scrambled siRNA kit (Stealth^TM^ RNAi Negative Control Duplexes, 12935–100; Invitrogen, San Diego, CA, USA) was used as the negative control. Transient transfection was performed using an MP-100 Microporator (Digital Biotechnology), according to the manufacturer’s instructions for optimization and usage. The plasmid expressing GFP-PTEN (plasmid #13039) and its control GFP (plasmid #40768) used in this study were purchased from Addgene (Cambridge, MA, USA). The plasmid expressing pcDNA-Keap1 (plasmid #62456) were purchased from Addgene (Cambridge, MA, USA). Its control pcDNA (Cat# 69864-3CN) used in this study were purchased from Merck (Darmstadt, Germany). After transfection, the cells were cultured for 24 h prior to the experiments.

### RNA Interference

Proteins were down-regulated using lentiviral expression of short hairpin RNA (shRNA) targeting the human PTEN shRNA construct (TRCN0000028992, containing the shRNA target sequence 5′-GCTAGAACTTATCAAACCCTT-3′ for mouse PTEN), human mTOR (TRCN0000038678 containing the following shRNA target sequence: 5′-GCATGGAAGAATACACCTGTA-3′), human Atg5 (TRCN0000151963 containing the following shRNA target sequence: 5′-CCTGAACAGAATCATCCTTAA -3′), and the luciferase shRNA construct (TRCN0000072247, containing the shRNA target sequence 5′-GAATCGTCGTATGCAGTGAAA-3′ for a negative control) were used to generate recombinant lentiviral particles (luciferase shRNA, shLuc). The shRNA constructs were purchased from the National RNAi Core Facility (Institute of Molecular Biology/Genomic Research Center, Academia Sinica, Taiwan). Lentiviruses were prepared, and the cells were infected according to previously described protocols^19^. In brief, the AS2 cells were transduced by lentivirus with the appropriate multiplicity of infection in a complete growth medium supplemented with polybrene (Sigma-Aldrich). After transduction for 24 h and puromycin (Calbiochem, San Diego, CA, USA) selection for 3 days, protein expression was monitored using a western blot analysis.

### Intracellular ROS assay

Intracellular oxidative stress was measured using dichlorodihydrofluorescein diacetate oxidation. Cells were exposed to 20 µM 5-(and-6)-chloromethyl -2’,7’-dichlorodihydrofluorescein diacetate, acetyl ester (CM-H_2_DCFDA) (Invitrogen) for 1 h. The cells were detected with the FL-1 channel (515–545 nm) by FACSCalibur. The data was analyzed using CellQuest Pro 4.0.2 software, and quantification was done using WinMDI 2.8 software. Small cell debris was excluded by gating on a forward scatter plot.

### qRT-PCR

Total cellular RNA was extracted using a Direct-zol RNA MiniPrep kit (Zymo Research, #R2050). The concentration of RNA was quantified by spectrophotometry at 260 nm (NanoDrop™ 2000/2000c Spectrophotometers, Thermo Scientific™). cDNA was synthesized from 1.0 μg of total RNA using the PrimeScriptTM RT reagent Kit (TaKaRa, #RR037A). The reaction mixture was incubated at 37 °C for 15 min and then at 85 °C for 5 s. The cDNA (0.2 μl) was added to the Fast SYBR Green MasterMix (Applied Biosystems™, # 4385612) in a total reaction volume of 20 μl. Oligonucleotide primers for human were MAP1LC3B (LC3B), 5′-AGCAGCATCC AACCAAAATC-3′ (forward) 5′-CTGTGTCCGT TCACCAACAG-3′ (reverse); SQSTM1, 5′-CACCTGTCTG AGGGCTTCTC-3′ (forward) 5′-CACACTCTCC CCAACGTTCT-3′ (reverse); ACTB, 5′-GGACTTCGAGCAAGAGATGG-3′ (forward) 5′-AGCACTGTGT TGGCGTACAG-3′ (reverse). A relative gene-expression quantification method was used to calculate the fold change of mRNA expression according to the comparative Ct method using ACTB for normalization. Final results were determined by the formula: 2^−(ΔCt sample−ΔCt control)^, where ΔCt values of the control and sample were determined by subtracting the Ct value of the target gene from the value of the housekeeping gene (ACTB). Data was represented as a ratio of the treated sample value to the control sample value.

### Immunofluorescence

Cells were fixed with 4% paraformaldehyde in PBS for 30 min and then permeabilized with permeabilization buffer (0.5% Triton-X100) for 15 min at room temperature. After that, cells were blocked with 1% BSA in PBS for 60 min at room temperature, and then stained with anti-LC3B antibody (GeneTex, #GTX127375) at 4 °C for overnight. Cells were incubated with Alexa488-conjugated secondary antibody (Life Technologies, #A11034) at room temperature for 1 h, and then the nuclei were stained with 0.5 μg/ml DAPI at roomtemperature for 10 min. Sample were observed by an immunofluorescence microscope (Olympus IX71).

### Statistical analysis

The values are provided as the means ± standard deviations (SDs). The groups were compared using a Student’s two-tailed unpaired *t* test; significance was set at a *P*-value of <0.05.

## Results

### High autophagy activity in lung cancer induces low Keap1 expression

**Fig. 1:**
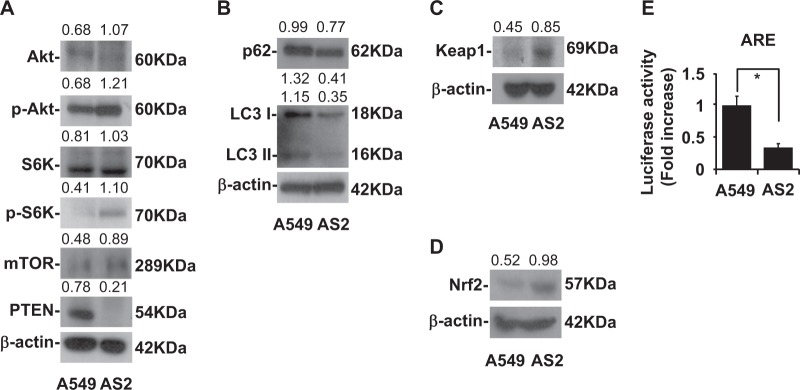
More dominant autophagy and ARE expression in A549 than in AS2 cells . **a** A representative western blot analysis showing the expression of the Akt, p-Akt, S6K, p-S6K, mTOR, PTEN in A549 and AS2 cells. β-actin was used as an internal control. The relative densities of the measured protein bands are also shown. **b** A representative western blot analysis showing the expression of the p62, LC3 I, LC3 II in A549 and AS2 cells. β-actin was used as an internal control. The relative densities of the measured protein bands are also shown. **c** A representative western blot analysis showing the expression of Keap1 in the A549 and AS2 cells. β-actin was used as an internal control. The relative densities of the measured protein bands are also shown. **d** A representative western blot analysis showing the expression of Nrf2 in the A549 and AS2 cells. β-actin was used as an internal control. The relative densities of the measured protein bands are also shown. **e** A luciferase activity analysis showing the activities of ARE in the A549 and AS2 cells. The mean luciferase activity of each stain is shown as the means ± SDs of three individual experiments. *P *<* 0.05

We examined several regulators of autophagy in lung cancer A549 and AS2 cells. Western blot analyses demonstrated lower expression of Akt and mTOR and higher expression of PTEN level in A549 cells as compared to the AS2 cells (Fig. 1a). We observed higher p62 and LC3 I and II levels in the A549 cells than in the AS2 cells (Fig. 1b). Lower Keap1 and Nrf2 levels were found in A549 cells as compared to AS2 cells (Fig. 1c, d). The A549 cells had higher ARE activity than the AS2 cells (Fig. 1e).

### PTEN overexpression induces resistance to VNR and high autophagy activity and inhibition of PTEN induces sensitive to VNR and low autophagy activity

**Fig. 2:**
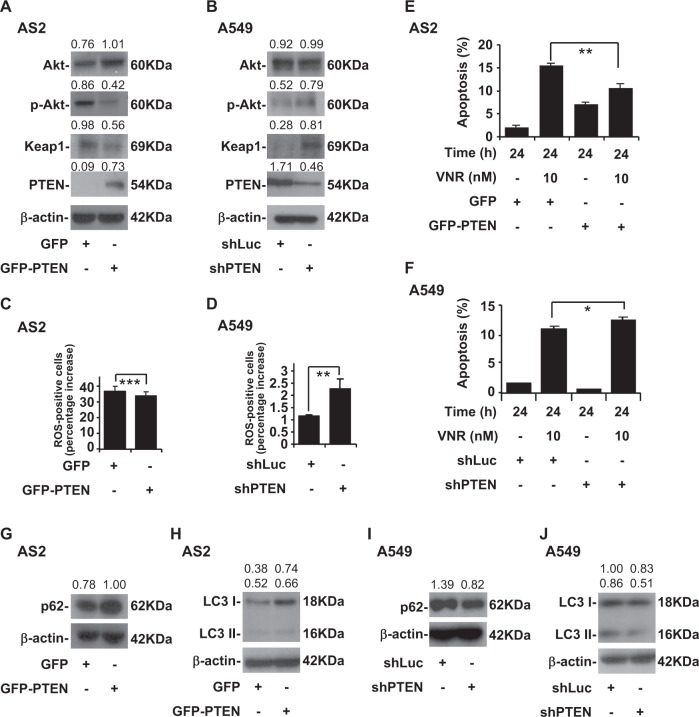
PTEN regulates autophagy activity followed by ROS level and VNR-induced apoptosis in AS2 and A549 cells. **a** A representative western blot analysis showing the expression of Akt, p-Akt, Keap1 and PTEN in the AS2 cells without or with the transfection of plasmids containing GFP-PTEN. GFP was used as a vector control. β-actin was used as an internal control. The relative densities of the measured protein bands are also shown. **b** A representative western blot analysis showing the expression of Akt, p-Akt, Keap1, PTEN in the A549 cells with shLuc and shPTEN. **c** CM-H_2_DCFDA staining, followed by a flow cytometric analysis, was used to determine the levels of ROS in AS2 cells transduced with GFP and GFP-PTEN. For the flow cytometric analyses, the percentages are the means ± SDs of three individual experiments. ***P *<* 0.001. **d** CM-H2DCFDA staining, followed by a flow cytometric analysis, was used to determine the levels of ROS in A549 cells with shLuc and shPTEN. For the flow cytometric analyses, the percentages are the means ± SDs of three individual experiments. **P < 0.01. **e** Nuclear PI staining and subsequent flow cytometric analysis determined cell apoptosis in VNR-treated AS2 cells transduced with GFP and GFP-PTEN. The percentages (%) of apoptotic cells are shown as the means ± SDs of three individual experiments. **P < 0.01. **f** Nuclear PI staining and subsequent flow cytometric analysis determined cell apoptosis in the VNR-treated A549 cells with shLuc and shPTEN. The percentages (%) of apoptotic cells are shown as the means ± SDs of three individual experiments. *P < 0.05. A representative western blot analysis showing the expression of p62 (**g**) and LC3 I and II (**h**) in AS2 cells transduced with GFP and GFP-PTEN. β-actin was used as an internal control. The relative densities of the measured protein bands are also shown. A representative western blot analysis showing the expression of p62 (**i**) and LC3 I and II (**j**) in the A549 cells with shLuc and shPTEN

Because AS2 cells lost PTEN, we next examined the effects of PTEN over-expression in AS2 cells. A western blot analysis showed over-expression of PTEN decreased p-Akt and Keap1 levels in the AS2 cells (Fig. 2a). Because the A549 cells had high PTEN expression, we next examined the role of blocking PTEN in our model. A western blot analysis showed PTEN knockdown with shRNA increased p-Akt and Keap1 levels in the A549 cells (Fig. 2b). CM-H_2_DCFDA staining, followed by a flow cytometric analysis, demonstrated that over-expression of PTEN significantly decreased the generation of intracellular ROS in the AS2 cells (*P* *<* 0.001) (Fig. 2c), and PTEN knockdown significantly increased the generation of intracellular ROS in the A549 cells (*P* < 0.01) (Fig. 2d). Nuclear PI staining, followed by flow cytometry, revealed that over-expression of PTEN in the AS2 cells contributed to lower VNR-induced apoptosis as compared to the AS2 cells (*P* *<* 0.01) (Fig. 2e), and PTEN knockdown in the A549 cells contributed to higher VNR-induced apoptosis than was the case in the A549 cells (*P* < 0.05) (Fig. 2f). A western blot analyses showed that PTEN over-expression in the AS2 cells resulted in higher p62 (Fig. 2g) and LC3 I and II levels (Fig. 2h) than was the case in the AS2 cells, and PTEN knockdown in the A549 cells had lower p62 (Fig. 2i) and LC3 I and II levels (Fig. 2j) than was the case in the A549 cells.

### Inhibition of mTOR induces resistant to VNR and high autophagy activity

**Fig. 3:**
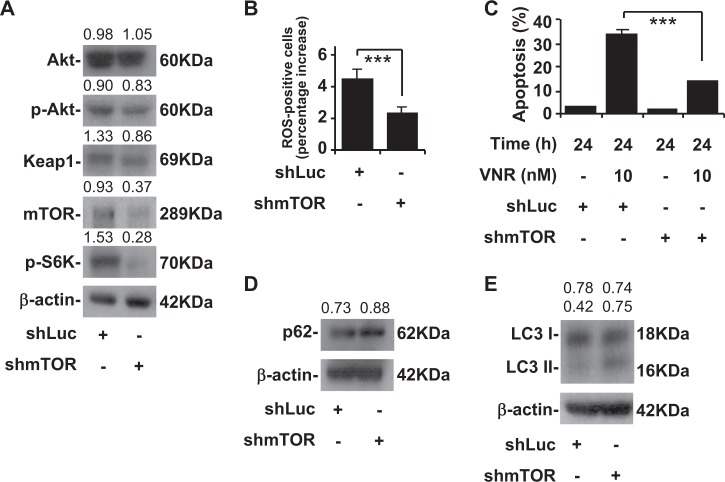
mTOR knockdown induces increased autophagy activity followed by diminished ROS and VNR-induced apoptosis in the AS2 cells. **a** A representative western blot analysis showing the expression of Akt, p-Akt, Keap1, mTOR and p-S6K in the AS2 cells with shLuc and shmTOR. β-actin was used as an internal control. The relative densities of the measured protein bands are also shown. **b** CM-H_2_DCFDA staining, followed by a flow cytometric analysis, was used to determine the levels of ROS in the AS2 cells with shLuc and shmTOR. For the flow cytometric analyses, the percentages are the means ± SDs of three individual experiments. ***P *<* 0.001. **c** Nuclear PI staining and a subsequent flow cytometric analysis determined cell apoptosis in VNR-treated AS2 cells with shLuc and shmTOR. The percentages (%) of apoptotic cells are shown as the means ± SDs of three individual experiments. ***P *<* 0.001. A representative western blot analysis showing the expression of p62 (**d**) and LC3 I and II (**e**) in the AS2 cells with shLuc and shmTOR. β-actin was used as an internal control. The relative densities of the measured protein bands are also shown

Because AS2 cells had high mTOR expression, we next examined the role of blocking mTOR in our model. A western blot analysis showed that mTOR knockdown with shRNA decreased p-Akt, Keap1, and p-S6K levels in the AS2 cells (Fig. 3a). CM-H_2_DCFDA staining, followed by a flow cytometric analysis, demonstrated that mTOR knockdown significantly decreased the generation of intracellular ROS in the AS2 cells (*P* *<* 0.001) (Fig. 3b). Nuclear PI staining, followed by flow cytometry, revealed that mTOR knockdown in the AS2 cells contributed to lower VNR-induced apoptosis than was the case for the AS2 cells (*P* *<* 0.001) (Fig. 3c). Western blot analyses showed that mTOR knockdown in the AS2 cells had higher p62 (Fig. 3d) and LC3 II levels (Fig. 3e) than was the case in the AS2 cells.

### Inhibition of Keap1 induces resistant to VNR and high autophagy activity and Keap1 overexpression induces sensitivity to VNR and low autophagy activity

**Fig. 4:**
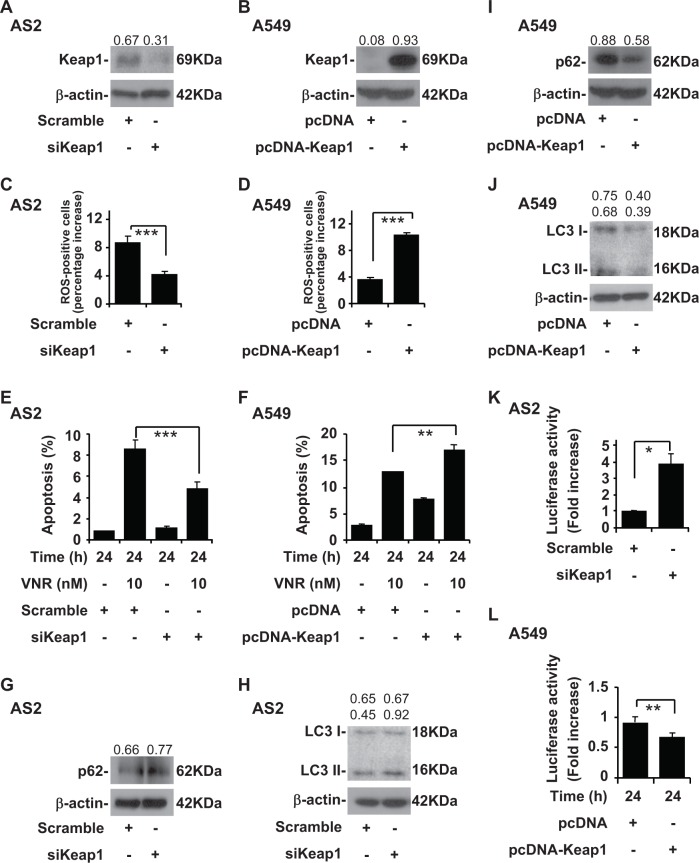
Keap1 regulates autophagy activity followed by ROS level and VNR-induced apoptosis in the AS2 and A549 cells. **a** A representative western blot analysis showing the expression of Keap1 in the AS2 cells with or without the transfection of plasmids containing siKeap1. β-actin was used as an internal control. The relative densities of the measured protein bands are also shown. **b** A representative western blot analysis showing the expression of Keap1 in the A549 cells with or without the transfection of plasmid expressing pcDNA-Keap1, where pcDNA was used as a vector control. **c** CM-H_2_DCFDA staining, followed by a flow cytometric analysis, was used to determine the levels of ROS in the AS2 cells with or without the transfection of plasmids containing siKeap1. For the flow cytometric analyses, the percentages are the means ± SDs of three individual experiments. ***P *<* 0.001. **d** CM-H2DCFDA staining, followed by a flow cytometric analysis, was used to determine the levels of ROS in the A549 cells transduced with pcDNA and pcDNA-Keap1. For flow cytometric analyses, the percentages are the means ± SDs of three individual experiments. ***P < 0.001. **e** Nuclear PI staining and a subsequent flow cytometric analysis determined cell apoptosis in the VNR-treated AS2 cells with or without the transfection of plasmids containing siKeap1. The percentages (%) of apoptotic cells are shown as the means ± SDs of three individual experiments. ***P *<* 0.001. **f** Nuclear PI staining and a subsequent flow cytometric analysis determined cell apoptosis in the VNR-treated A549 cells transduced with pcDNA and pcDNA-Keap1. The percentages (%) of apoptotic cells are shown as the means ± SDs of three individual experiments. **P < 0.01. A representative western blot analysis showing the expression of p62 (**g**) and LC3 I and II (**h**) in the AS2 cells with or without the transfection of plasmids containing siKeap1. β-actin was used as an internal control. The relative densities of the measured protein bands are also shown. A representative western blot analysis showing the expression of p62 (**i**) and LC3 I and II (**j**) in the A549 cells transduced with pcDNA and pcDNA-Keap 1. **k** The luciferase activity analysis showing the expression antioxidant response element of in AS2 with or without the transfection of plasmids containing siKeap1. The mean luciferase activity (fold increase) of each stain is shown as the means ± SDs of three individual experiments, *P < 0.05. **l** The luciferase activity analysis showing the expression antioxidant response element of in A549 transduced with pcDNA and pcDNA-Keap1. The mean luciferase activity (fold increase) of each stain is shown as the means ± SDs of three individual experiments, **P < 0.01

Because AS2 cells had high Keap1 expression, we next examined the role of blocking Keap1 in our model. A western blot analysis showed Keap1 knockdown with siRNA decreased Keap1 levels in the AS2 cells (Fig. 4a). Because A549 cells had low Keap1 expression, we next examined the over-expression of Keap1 in the A549 cells. A western blot analysis showed over-expression of Keap1 increased Keap1 levels in the A549 cells (Fig. 4b). CM-H_2_DCFDA staining, followed by a flow cytometric analysis, demonstrated that Keap1 knockdown significantly decreased the generation of intracellular ROS in the AS2 cells (*P* *<* 0.001) (Fig. 4c), and over-expression of Keap1 significantly increased the generation of intracellular ROS in the A549 cells (*P* < 0.001) (Fig. 4d). Nuclear PI staining, followed by flow cytometry, revealed that Keap1 knockdown in the AS2 cells contributed to lower VNR-induced apoptosis than was the case for the AS2 cells (*P* *<* 0.001) (Fig. 4e), and over-expression of Keap1 in the A549 cells contributed to higher VNR-induced apoptosis than in the A549 cells (*P* < 0.01) (Fig. 4f). Western blot analyses showed that the Keap1 knockdown in the AS2 cells had higher p62 (Fig. 4g) and LC3 I and II levels (Fig. 4h) than was the case in the AS2 cells, and over-expression of Keap1 in the A549 cells had lower p62 (Fig. 4i) and LC3 I and II levels (Fig. 4j) than was the case in the A549 cells. A luciferase activity analysis revealed that Keap1 knockdown in the AS2 cells had amplified ARE in the luciferase activity analysis (Fig. 4k), and decreased ARE in Keap1 overexpression in the A549 cells (Fig. 4l).

### Inhibition of ATG5 induces sensitivity to VNR and low autophagy activity, and blocking autophagy regulates VNR sensitivity

**Fig. 5:**
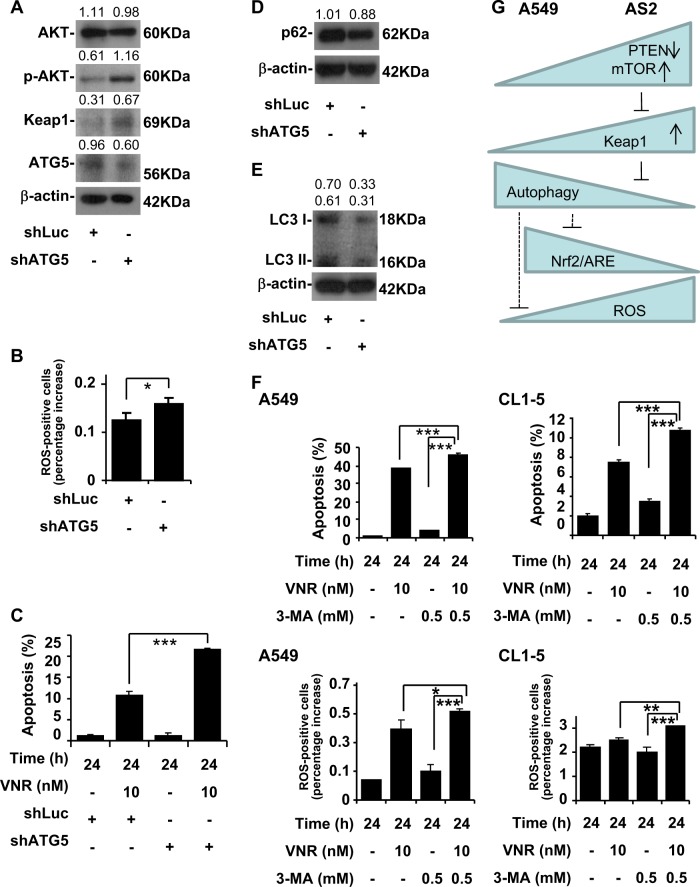
Blocking autophagy induces the amplification of ROS and VNR-induced apoptosis in the A549 and CL1-5 cells. **a** A representative western blot analysis showing the expression of Akt, p-Akt, Keap1, ATG5 in the A549 cells with shLuc and shATG5. β-actin was used as an internal control. The relative densities of the measured protein bands are also shown. **b** CM-H_2_DCFDA staining, followed by a flow cytometric analysis, was used to determine the levels of ROS in A549 cells with shLuc and shATG5. For the flow cytometric analyses, the percentages are the means ± SDs of three individual experiments. *P *<* 0.5. **c** Nuclear PI staining and subsequent flow cytometric analysis determined cell apoptosis in the VNR-treated A549 cells with shLuc and shATG5. The percentages (%) of apoptotic cells are shown as the means ± SDs of three individual experiments. ***P *<* 0.001. A representative western blot analysis showing the expression of p62 (**d**) and LC3 I and II (**e**) in A549 cells with shLuc and shATG5. β-actin was used as an internal control. The relative densities of the measured protein bands are also shown. **f** Nuclear PI staining and subsequent flow cytometric analysis determined cell apoptosis in the VNR-treated A549 and CL1-5 cells with or without autophagy inhibitor 3-MA. The percentages (%) of apoptotic cells are shown as the means ± SDs of three individual experiments. ***P < 0.001. CM-H2DCFDA staining, followed by a flow cytometric analysis, was used to determine the levels of ROS in the VNR-treated A549 and CL1-5 cells with or without autophagy inhibitor 3-MA. For the flow cytometric analyses, the percentages are the means ± SDs of three individual experiments. *P < 0.05, **P < 0.01, ***P < 0.001. **g** A hypothetic model of this work. Targeting the regulators of autophagy modulates Keap1-mediated ARE activity, ROS generation, and VNR-induced apoptosis

Because A549 cells had high ATG5 expression, we next examined the role of blocking ATG5 in our model. A western blot analysis showed ATG5 knockdown with shRNA increased p-Akt and Keap1 levels in the A549 cells (Fig. 5a). CM-H_2_DCFDA staining, followed by flow cytometric analysis, demonstrated that ATG5 knockdown significantly increased the generation of intracellular ROS in the A549 cells (*P* *<* 0.05) (Fig. 5b). Nuclear PI staining, followed by flow cytometry, revealed that ATG5 knockdown in the A549 cells contributed to higher VNR-induced apoptosis than in the A549 cells (*P* *<* 0.001) (Fig. 5c). Western blot analyses showed that ATG5 knockdown in the A549 cells had lower p62 (Fig. 5d) and LC3 I and II levels (Fig. 5e) than in the A549 cells. Blocking autophagy with 3-MA induced increased ROS accumulation and VNR-induced apoptosis in both the A549 and CL1-5 cells (Fig. 5f).

## Discussion

As summarized in Fig. 5g, the pilot study demonstrated a VNR-sensitive strategy in lung adenocarcinoma cells, by which regulators of autophagy modulated Keap1-mediated ROS generation and VNR-induced apoptosis. We summarized some regulators of autophagy, including autophagy-suppressing factors (class I PI3K/Akt/mTOR) and autophagy-promoting factors (PTEN)^8^.

Bcl-2 and autophagy protein Beclin 1 also inhibit autophagy-associated cell death^20^, but the system was not significant in our model (data not shown). Our study revealed similar results to those previously published indicating that the A549 cells had lower p-Akt and p-S6K and higher PTEN and LC3 I and II levels than the AS2 cells^21^. The manipulation of PTEN and mTOR in the AS2 cells allowed further characterization of their roles in diminished Akt and Keap1, amplified autophagic p62, LC3 expression accompanied with decreased ROS, and VNR-induced apoptosis. Therefore, PTEN and mTOR were regulators of autophagy, which could have modulated the AS2-treated VNR sensitivity. Previous publications have also concluded that upregulated autophagy responds to stress induced by chemotherapy and radiation^22^.

In addition, AS2 cells were modified with Keap1 knockdown, which demonstrated increased autophagy activities with reduced ROS and VNR-induced apoptosis. This is the first study to our knowledge to demonstrate that Keap1 is a key point of connection between regulators of autophagy and VNR sensitivity. Keap1 inhibits Nrf2 activity constitutively under general conditions, but under stress, Keap1-mediated proteasomal degradation of Nrf2 is suppressed, which induces Nrf2 nuclear accumulation and transcription that enhances cell survival^23,24^. Loss of Keap1 function induces constitutive activation of Nrf2-mediated gene expression against chemotherapeutic agents in lung cancer^25^. Therefore, modulation of the regulators of autophagy and Keap1 function are the essential targets for eliminating drug resistance.

Reversely, PTEN knockdown in the A549 cells induced declined p62 and LC3 expression accompanied with accumulated ROS and VNR-induced apoptosis. p62 overexpression significantly diminishes the half-life of Keap1, and p62 knockdown increases it^26^. p62 sequesters Keap1 into the autophagosomes and induces activation of the Nrf2 signaling pathway^15^. In Atg5-deficient A549 cells, the levels of intracellular and mitochondrial ROS and the number of mitochondria are significantly increased^21^. Modifying A549 cells with Atg5 knockdown increases Akt activity followed by Keap1-mediated ROS generation and VNR-induced apoptosis. Autophagy-deficient cells have defective mitochondrial clearance, resulting in increased ROS production^19^. Previous researchers have frequently induced autophagy deficiency with Atg5 knockdown because it plays an essential role in the initiation of autophagy^27^. Keap1 overexpression also reduces autophagy activity with accumulated ROS and VNR-induced apoptosis in A549 cells because Keap1 overexpression may enhance Nrf2 ubiquitylation. Previous publications have demonstrated that activating Nrf2 increases its nuclear translocation for hypoxia or ROS in A549 cells^28,29^. ROS/Autophagy/Nrf2 pathway has been shown to regulate radiation-resistance in A549 lung cancer cells^30^.

Because autophagy plays an important role in chemotherapy resistance, we hypothesized that regulators of autophagy would modulate VNR sensitivity through Keap1-mediated ROS generation. In our model, modifying PTEN, mTOR, and Atg5 indeed modulate p62 and LC3 expression and Keap1 activity followed by ROS level and VNR-induced apoptosis. On May 30, 2007, the U. S. Food and Drug Administration granted approval for temsirolimus, an mTOR inhibitor, for the treatment of advanced renal cell carcinoma. PTEN and mTOR were proven to be significant regulators of autophagy in our study, but further studies are needed to provide enough evidence for clinical practice of target therapy in lung cancer. In addition, Keap1 expression also regulated autophagy activity, ROS levels and VNR sensitivity. Declining levels of ARE were demonstrated in Keap1 overexpression A549 cells, and we posited that Nrf2 did not translocate to the nucleus and activate transcription for further survive signaling. Reversely, enhanced ARE was noticed in Keap1 knockdown AS2 cells, which means that survival signaling should be transmitted to modulate autophagy and ROS generation. Blocking autophagy with 3-MA also caused increased ROS accumulation and VNR-induced apoptosis in the A549 and CL1-5 cells. A phase III clinical trial for glioblastoma multiforme patients, combining radiation, carmustine, and chloroquine (autophagy inhibitor) demonstrated a better survival rate than a placebo arm^31^. However, the results were not statistically significant because of the limited sample size^32^. Several publications discussing Keap1 metabolism or blocking with small molecules^33^ have been explored, and Keap1 degradation by autophagy was p62-dependent^17^. We concluded that targeting in autophagy or Keap1 must be the next essential therapeutic development for chemotherapy resistance in lung cancer.

## Electronic supplementary material

supplemental data

flowcytometric raw data for reviewer
