# Supplementary material for: Autophagy regulates vinorelbine sensitivity due to continued Keap1-mediated ROS generation in lung adenocarcinoma cells
Source: Cell Death Discov. 2018 Sep 12;4:96. doi: 10.1038/s41420-018-0098-6 (PMC6135768; doi:10.1038/s41420-018-0098-6)
Supplement: Supplementary file 1 — supplemental data [file 41420_2018_98_MOESM1_ESM.ppt]

## Slide 1
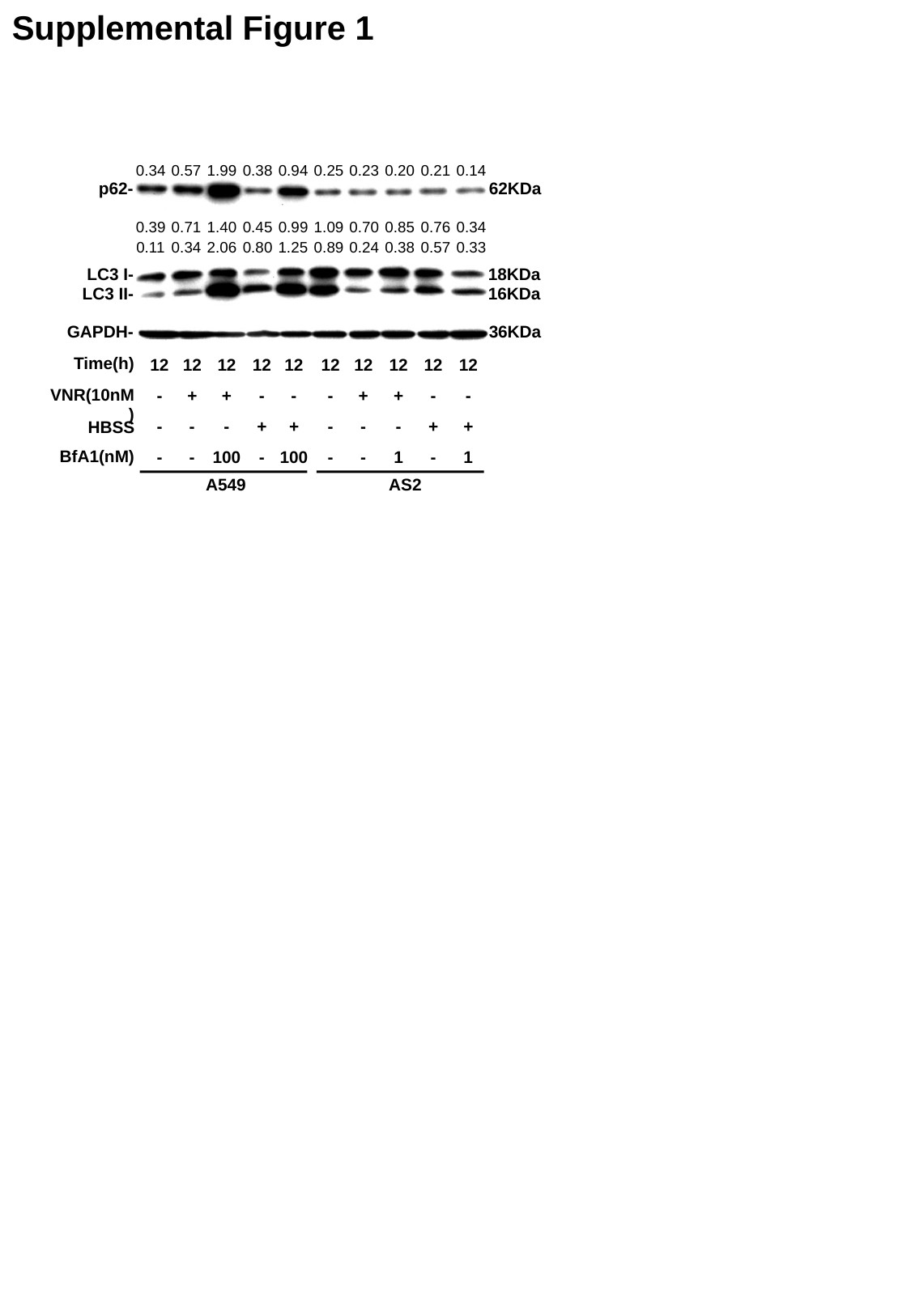

Supplemental Figure 1
0.34
0.57
1.99
0.38
0.94
0.25
0.23
0.20
0.21
0.14
p62-
62KDa
0.39
0.71
1.40
0.45
0.99
1.09
0.70
0.85
0.76
0.34
0.11
0.34
2.06
0.80
1.25
0.89
0.24
0.38
0.57
0.33
LC3 I-
18KDa
LC3 II-
16KDa
GAPDH-
36KDa
Time(h)
12
-
-
-
12
+
-
-
12
+
-
100
12
-
+
-
12
-
+
100
12
-
-
-
12
+
-
-
12
+
-
1
12
-
+
-
12
-
+
1
VNR(10nM)
HBSS
BfA1(nM)
A549
AS2

## Slide 2
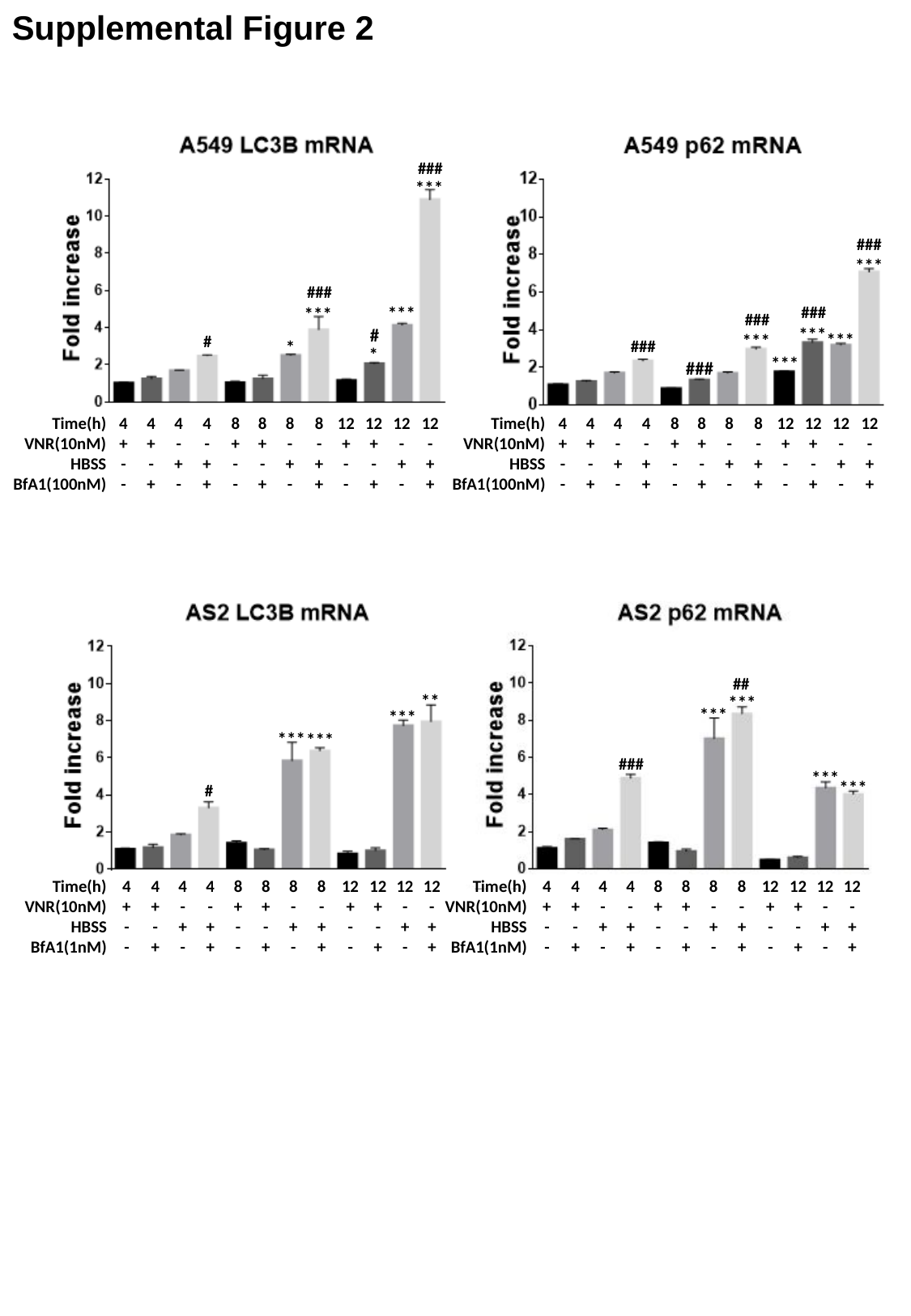

Supplemental Figure 2
###
***
###
***
###
***
###
***
###
***
#
***
***
#
*
###
*
***
###
Time(h)
VNR(10nM)
HBSS
BfA1(100nM)
4
+
-
-
4
+
-
+
4
-
+
-
4
-
+
+
8
+
-
-
8
+
-
+
8
-
+
-
8
-
+
+
12
+
-
-
12
+
-
+
12
-
+
-
12
-
+
+
Time(h)
VNR(10nM)
HBSS
BfA1(100nM)
4
+
-
-
4
+
-
+
4
-
+
-
4
-
+
+
8
+
-
-
8
+
-
+
8
-
+
-
8
-
+
+
12
+
-
-
12
+
-
+
12
-
+
-
12
-
+
+
##
**
***
***
***
***
***
###
***
***
#
Time(h)
VNR(10nM)
HBSS
BfA1(1nM)
4
+
-
-
4
+
-
+
4
-
+
-
4
-
+
+
8
+
-
-
8
+
-
+
8
-
+
-
8
-
+
+
12
+
-
-
12
+
-
+
12
-
+
-
12
-
+
+
Time(h)
VNR(10nM)
HBSS
BfA1(1nM)
4
+
-
-
4
+
-
+
4
-
+
-
4
-
+
+
8
+
-
-
8
+
-
+
8
-
+
-
8
-
+
+
12
+
-
-
12
+
-
+
12
-
+
-
12
-
+
+

## Slide 3
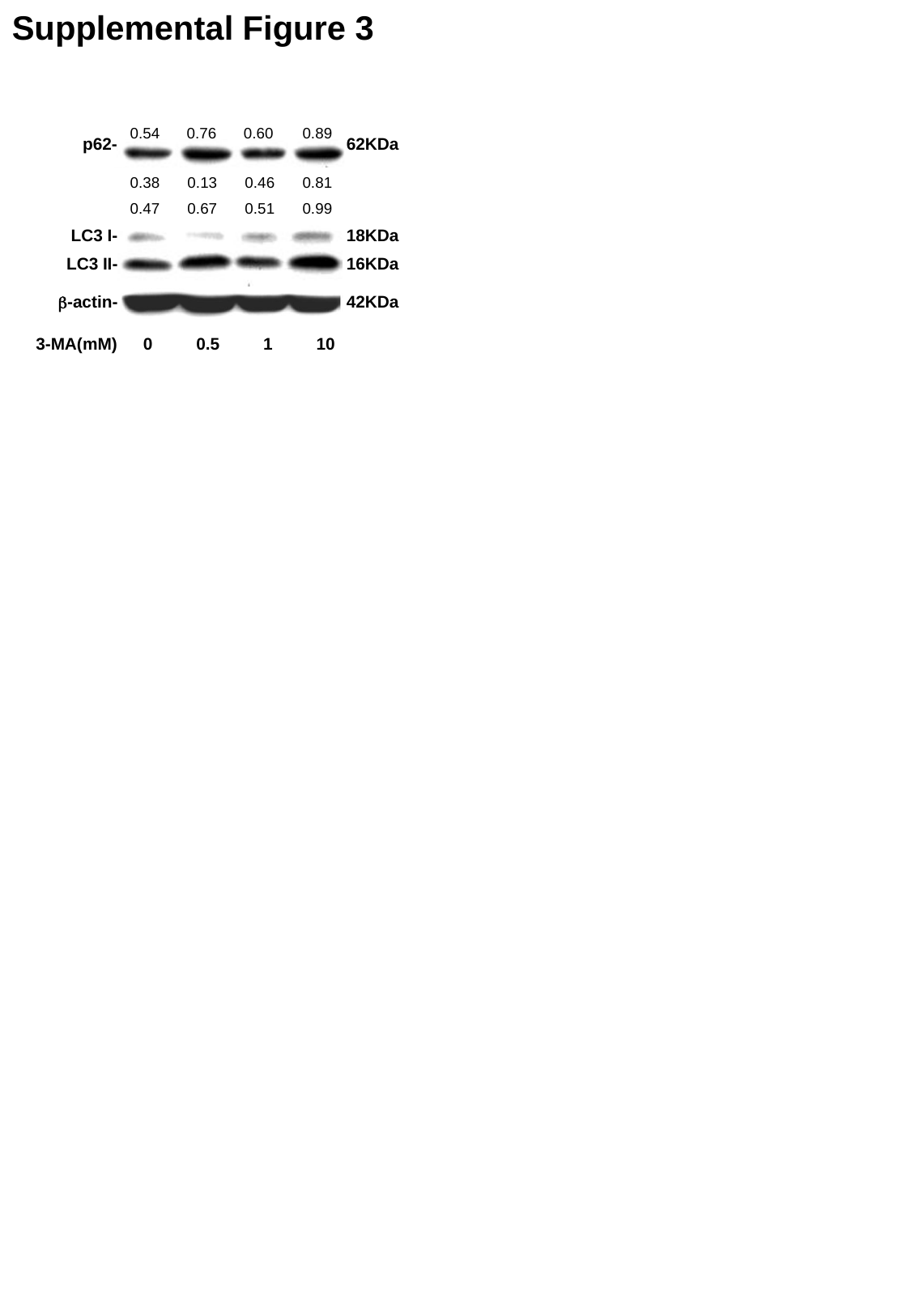

Supplemental Figure 3
0.54
0.76
0.60
0.89
p62-
62KDa
0.38
0.13
0.46
0.81
0.47
0.67
0.51
0.99
LC3 I-
18KDa
LC3 II-
16KDa
β-actin-
42KDa
3-MA(mM)
0
0.5
1
10

## Slide 4
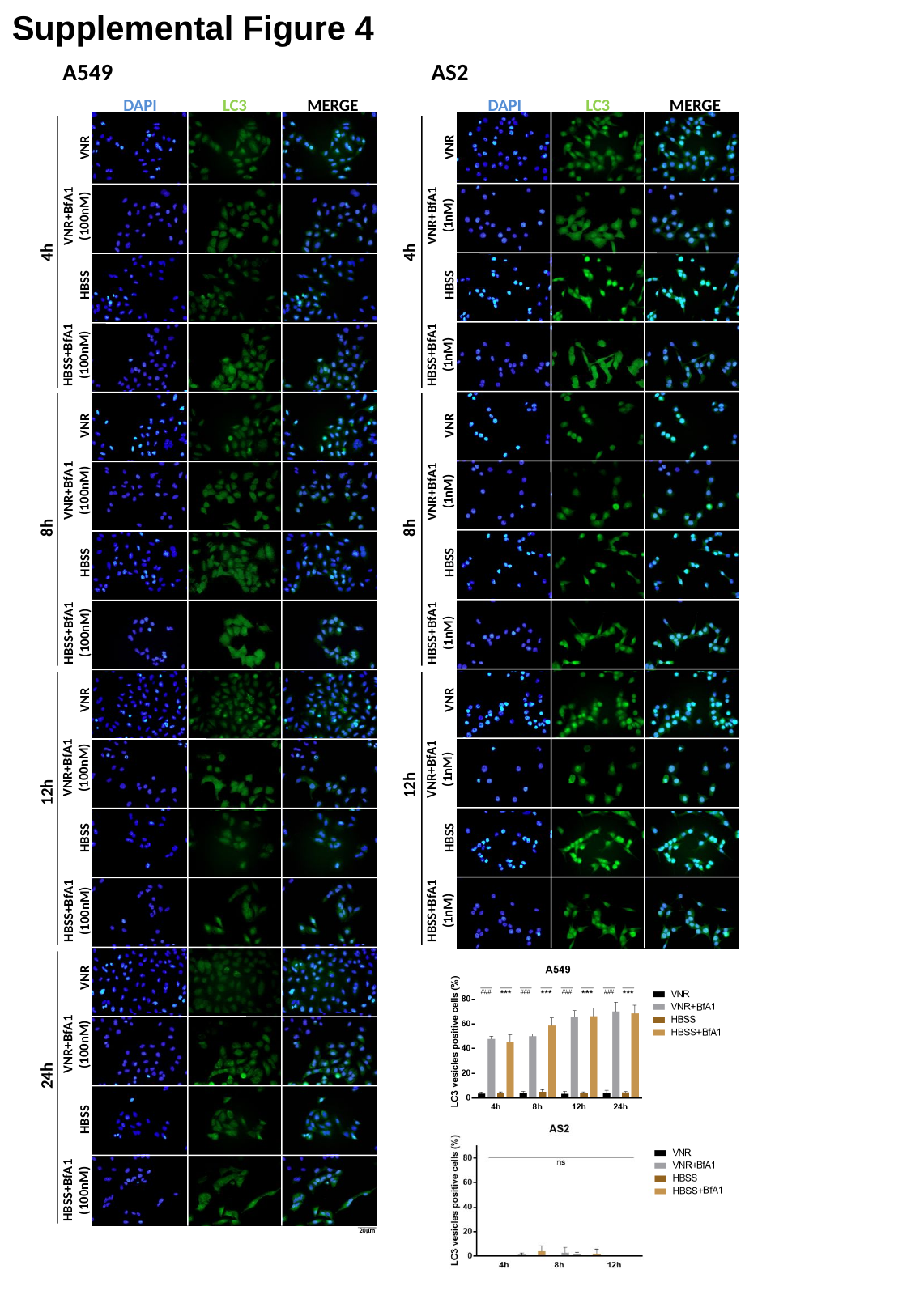

Supplemental Figure 4
A549
AS2
DAPI
LC3
MERGE
DAPI
LC3
MERGE
VNR
VNR
VNR+BfA1
(100nM)
VNR+BfA1
(1nM)
4h
4h
HBSS
HBSS
HBSS+BfA1
(100nM)
HBSS+BfA1
(1nM)
VNR
VNR
VNR+BfA1
(100nM)
VNR+BfA1
(1nM)
8h
8h
HBSS
HBSS
HBSS+BfA1
(100nM)
HBSS+BfA1
(1nM)
VNR
VNR
VNR+BfA1
(100nM)
VNR+BfA1
(1nM)
12h
12h
HBSS
HBSS
HBSS+BfA1
(100nM)
HBSS+BfA1
(1nM)
20μm
VNR
VNR+BfA1
(100nM)
24h
HBSS
HBSS+BfA1
(100nM)
20μm
