# Supplementary material for: Autophagy regulates vinorelbine sensitivity due to continued Keap1-mediated ROS generation in lung adenocarcinoma cells
Source: Cell Death Discov. 2018 Sep 12;4:96. doi: 10.1038/s41420-018-0098-6 (PMC6135768; doi:10.1038/s41420-018-0098-6)
Supplement: Supplementary file 2 — flowcytometric raw data for reviewer [file 41420_2018_98_MOESM2_ESM.pptx]

## Slide 1
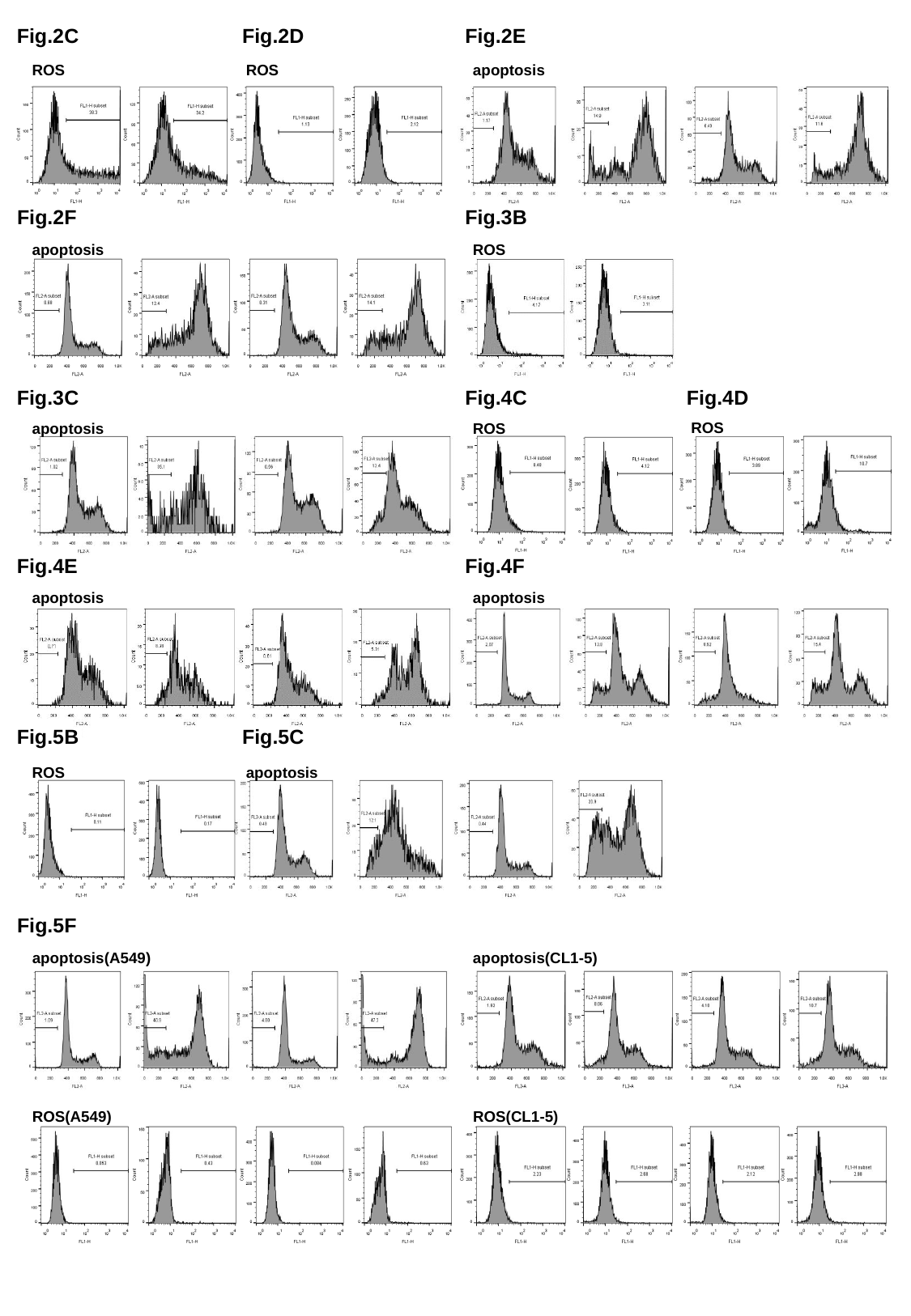

Fig.2C
Fig.2D
Fig.2E
ROS
ROS
apoptosis
Fig.2F
Fig.3B
apoptosis
ROS
Fig.3C
Fig.4C
Fig.4D
ROS
apoptosis
ROS
Fig.4E
Fig.4F
apoptosis
apoptosis
Fig.5B
Fig.5C
ROS
apoptosis
Fig.5F
apoptosis(A549)
apoptosis(CL1-5)
ROS(A549)
ROS(CL1-5)
